# Krankenhausmedizinische Interventionen in der neurologischen Anschlussrehabilitation

**DOI:** 10.1007/s00115-020-01021-9

**Published:** 2020-10-30

**Authors:** Stefan Knecht, Venus Koushk Jalali, Tobias Schmidt-Wilcke, Bettina Studer

**Affiliations:** grid.411327.2 0000 0001 2176 9917 St. Mauritius Therapieklinik Meerbusch, Institut für Klinische Neurowissenschaften und Medizinische Psychologie der Heinrich Heine Universität Düsseldorf, Düsseldorf, Deutschland

**Keywords:** Neurologie, Rehabilitation, Komplikationen, Gesundheitssektoren, Integrierte Neurorehabilitation, Neurology, Rehabilitation, Complications, Health care sectors, Integrated neurorehabilitation

## Abstract

Behandlungen in einem Krankenhaus unterscheiden sich von Behandlungen in einer Rehabilitationsklinik rechtlich dadurch, dass den Patienten im Krankenhaus jederzeitige Hilfe durch Ärzte und anderes qualifiziertes Personal zur Verfügung stehen muss – in der Rehabilitationsklinik hingegen nicht. Seit der Abfassung der zugehörigen Sozialgesetze vor über 30 Jahren werden mehr akutmedizinische Interventionen durchgeführt und die Zahl der Älteren in der Bevölkerung hat zugenommen. Infolgedessen sind Patienten heute älter und multimorbider und dadurch komplikationsgefährdeter. Dies gilt insbesondere für die postakute neurologische Versorgung. Deswegen sind die ursprünglichen Rahmenkonzepte für neurologische Rehabilitationsbehandlung fragwürdig geworden. Wir untersuchten daher prospektiv, wie häufig Patienten in der neurologischen Anschlussrehabilitation akute Komplikationen entwickelten und sofortiger Hilfe durch qualifiziertes Personal bedurften. Wir fanden unter 759 innerhalb einer sechsmonatigen Beobachtungsperiode behandelten Patienten 602 krankenhausmedizinische Komplikationen (Stürze, akute Harnwegsinfekte, Fieber anderer Art, *Clostridium-difficile*-Diarrhöen, Pneumonien, respiratorische Insuffizienz, Septitiden, epileptische Anfälle und Herzrhythmusstörungen). Insgesamt musste so in der untersuchten Einrichtung im Mittel mehr als dreimal pro Tag akutmedizinisch interveniert werden. Wir schlussfolgern, dass neurologische Anschlussrehabilitation dem bisherigen sozialgesetzlichen Rahmen entwachsen ist und Krankenhausbehandlung umfasst.

## Abstract (en)

Treatment in hospitals differs from treatment in rehabilitation centers from a legal perspective because German law mandates that in hospitals physicians and other qualified personnel must be on duty at all times. This is not required for inpatient rehabilitation centers. Since this Act was passed more than 30 years ago, more acute medical interventions are now carried out and the number of older people in the population has increased. As a result patients are nowadays older, more multimorbid and therefore have a greater risk for medical complications. This is especially true for postacute neurological care. For this reason, the original legal framework for neurological rehabilitation treatment has become questionable. Therefore, we prospectively tested how often patients in inpatient neurorehabilitation suffer from complications that require immediate attention by qualified personnel. In 759 patients observed over a period of 6 months we found 602 complications requiring immediate interventions by physicians (e.g. falls, urinary tract infections, other forms of fever, diarrhea associated with* Clostridium difficile*, pneumonia, respiratory insufficiency, sepsis, epileptic seizures and arrhythmia). On average at least three acute care interventions occurred per day at the facility examined. We conclude that neurological inpatient rehabilitation has outgrown its legal foundations and now incorporates hospital care.

## Hintergrund

Die grundgesetzliche Verpflichtung des Staates zur Gesundheitsfürsorge wird in Deutschland durch die Sozialgesetzgebung geregelt [10]. Diese sieht eine Unterscheidung von Krankenhäusern und Rehabilitationskliniken vor. Aufgabe von Krankenhäusern ist, Krankheiten der Patienten zu erkennen, zu heilen und ihre Verschlimmerung zu verhüten. Aufgabe von Vorsorge- oder Rehabilitationseinrichtungen ist auch, Krankheit zu heilen und ihre Verschlimmerung zu verhüten, aber zusätzlich erzielte Behandlungserfolge zu sichern, auch mit dem Ziel, eine drohende Behinderung oder Pflegebedürftigkeit abzuwenden. In Krankenhäusern muss zur Erfüllung der dortigen Aufgaben jederzeit ärztliches und anderes geschultes Personal verfügbar sein. Von Rehabilitationskliniken wird hingegen nur verlangt, dass sie fachlich-medizinisch unter ständiger ärztlicher Verantwortung stehen. Eine durchgehende Anwesenheit von ärztlichem und anderem geschulten Personal ist nicht gefordert, festgelegt im Sozialgesetzbuch (SGB) V, § 107. Dieser Gesetzespassus ist der vielleicht augenfälligste Ausdruck von Bemühungen im Nachkriegsdeutschland, Patientenversorgung unter Bezug auf historische Strukturen (Hospitäler gegenüber Kureinrichtungen) organisatorisch und regulatorisch zu sektorieren.

Dies gilt auch für die neurologische Versorgungskette. Zur Erinnerung: Sie lässt sich einteilen in die initiale Akutbehandlung mit allfälliger, nachfolgender neurologisch-neurochirurgischer Frührehabilitation im Krankenhaus. Danach haben selbsthilfeeingeschränkte Patienten mit Erholungschancen Anspruch auf eine Anschlussrehabilitation in einer neurologischen Rehabilitationsklinik. Diese wird abhängig von den funktionellen Einschränkungen nach der Bundesarbeitsgemeinschaft für Rehabilitation eingeteilt in die Phasen B bis F [1].

Im Gegensatz zur meist in Krankenhaussettings durchgeführten Frührehabilitation gilt in der weiteren postakuten Versorgung, also in der Anschlussrehabilitation, bis heute noch die erwähnte sozialgesetzliche Vorgabe, dass diese Rehabilitationskliniken nicht jederzeitig ärztliches und anderes qualifiziertes Personal vorhalten müssen. Diese sozialrechtliche Sektorentrennung ist jedoch über 30 Jahre alt und geprägt von einer gänzlich anderen Versorgungsrealität als heute. Deutschland war jünger und gesünder. Ferner gab es z. B. nach einem Schlaganfall keine systematische Versorgung in spezialisierten Einheiten, sodass die Krankenhausmortalität durch Hirnödem, Thrombosen oder Pneumonien hoch war. Wer dann nach einer mehr als doppelt so langen Krankenhausverweildauer wie heute in eine neurologische Anschlussrehabilitation kam, hatte schon eine gewisse Selektion durchlaufen.

Heute hingegen überleben Menschen schwerere Schlaganfälle und andere Erkrankungen trotz höheren Alters und Multimorbidität. Denn die medizinische Akutversorgung ist besser und intensiver geworden. Wir haben fast flächendeckend Stroke-Units und versorgen mehr als dreimal so viel Patienten intensivmedizinisch wie vor 30 Jahren [13]. Zusätzlich ist die Bevölkerung gealtert. Die Zahl der über 67-Jährigen ist heute doppelt so hoch wie vor 30 Jahren (16 gegenüber 8,4 Mio.; [13]). Und die heutige anteilige Lebenszeit mit Multimorbidität ist mehr als doppelt so hoch wie damals (30 % gegenüber 15 %; [14]). Entsprechend älter und kränker sind mittlerweile die Patienten bei Beginn einer neurologischen Anschlussrehabilitation [6]. Daher fragt es sich, ob die sozialgesetzlichen Konzepte der 1980er-Jahre noch zum heutigen Versorgungsgeschehen in der neurologischen Anschlussrehabilitation passen. Deswegen untersuchten wir an einer großen neurologischen Rehabilitationsklinik, wie häufig trotz systematischer Maßnahmen zur Komplikationsabwendung [5] unerwartete und jederzeitige ärztliche Interventionen nötig werden und damit sozialgesetzlich Kriterien gegeben sind, die Krankenhausbehandlung definieren.

## Methoden

### Definitionen und Operationalisierung

Zur Definition von krankenhausmedizinischen Interventionen orientierten wir uns an den sozialgesetzlichen Vorgaben (§§ 39, 40, 107 SGB V). Das SGB V definiert unter § 107 Abs. 1,3 Krankenhäuser als Einrichtungen, die „mit Hilfe von jederzeit verfügbarem ärztlichem, Pflege‑, Funktions- und medizinisch-technischem Personal darauf eingerichtet sind, vorwiegend durch ärztliche und pflegerische Hilfeleistung Krankheiten der Patienten zu erkennen, zu heilen, ihre Verschlimmerung zu verhüten“.

Eine jederzeitige Verfügbarkeit von ärztlichem, Pflege‑, Funktions- und medizinisch-technischem Personal ist dann notwendig, wenn bei Patienten Situationen wahrscheinlich sind, die sofort, zu Tages- oder Nachtzeiten, und unabhängig von anderen Verpflichtungen des Personals beherrscht werden müssen. Dies entspricht dem klinischen Konzept von Komplikationen, die wir als Ereignisse definierten, welche unmittelbarer ärztlicher Abklärung bedürfen:

1. Komplikationen, die von behandelnden Ärzten zum Zwecke der Koordination mit Diensthabenden und Nachbehandelnden in der
  - elektronischen klinischen Verlaufsdokumentation festgehalten oder
  - im wochentäglichen Frühbesprechung oder in der Wochenendbesprechung berichtet wurden,
2. neues Ansetzen eines Antibiotikums während des Rehabilitationsaufenthaltes,
3. Stürze, welche zu einer sofortigen ärztlichen Evaluation und allfälligen Weiterversorgung führten,
4. alle Fälle, die vom Notfallteam unseres Neurorehabilitationszentrums protokolliert wurden,
5. notfällige Verlegungen basierend auf der Dokumentation von Entlassungs‑/Verlegungsgrund nach § 301 Abs. 3 SGB V („Verlegung in ein anderes Krankenhaus“, „Verlegung von Reha in Krankenhaus“ und „Verlegung mit folgender Rückverlegung“) und
6. Todesfälle.

Ereignisse, die in mehreren dieser Dokumentationsquellen/-kategorien auftauchten, wurden nur einmal gezählt. Nicht gewertet wurden geplante Operationen, wie die Anlage eines suprapubischen Blasenkatheters oder einer perkutanen endoskopischen Gastrostomiesonde, obwohl diese normalerweise als Krankenhausleistung erbracht werden.

### Datenerhebung

Eingeschlossen wurden alle im September 2018 bis Februar 2019 in unserer neurologischen Rehabilitationsklinik behandelten Patienten, die ihr Einverständnis zu einer anonymisierten Auswertung ihrer Daten schriftlich bestätigt hatten (*n* = 759). Die Behandlungen (nach § 40 SBG V) liefen in über 95 % der Fälle zulasten der Krankenversicherung und in unmittelbarem Anschluss an eine vorangegangene Krankenhausbehandlung. Die Erhebung fand in der St. Mauritius Therapieklinik Meerbusch statt. Diese Einrichtung hat als integriertes Versorgungszentrum neben 270 (klassischen) Rehabilitationsbetten auch eine Krankenhausabteilung mit 30 Intensivbetten. Die gegenwärtige Auswertung bezog sich auf die Rehabilitationsbetten; Komplikationen in der Krankenhausabteilung wurden nicht gezählt.

Definitionen und Handhabung der Neurorehabilitationsphasen (B bis F) nach der Bundesarbeitsgemeinschaft für Rehabilitation [1] sind in Bundesländern variabel und zwischen Kostenträgern umstritten. In der hiesigen Beobachtungsstudie wurden so den Rehaphasen B bis D zugeordnete Patienten eingeschlossen. Im Weiteren benutzen wir den Begriff neurologische Anschlussrehabilitation, um die Rehabilitation im unmittelbaren Anschluss an die initiale und teilweise frührehabilitative Versorgung im Akutkrankenhaus zu beschreiben. Patienten werden jedoch vor allem nach dem Grad der Pflegeabhängigkeit charakterisiert, gemessen per Barthel-Index [9]. Um die Gesamtmorbidität der Patienten zu charakterisieren, bestimmten wir zudem für jeden Patienten den Charlson-Komorbiditätsindex unter Verwendung der Gewichtung von Quan und Kollegen [11]. Der Charlson-Komorbiditätsindex berücksichtigt Faktoren wie koronare Herzkrankheit, Herzinsuffizienz, Diabetes, Lebererkrankungen, Nierenerkrankungen, rheumatische Erkrankungen, HIV/AIDS, chronische Lungenerkrankung, Leukämie, maligner Tumor, Alkoholerkrankung und Demenz. Zudem schätzen wir das Ein-Jahres-Mortalitätsrisiko der Patienten anhand des gewichteten Charlson-Komorbiditätsindex, des Alters und der beobachteten Mortalitäten in einer großen dänischen Registerstudie [12].

Zur Bestimmung der Inzidenzen wurden alle im Beobachtungszeitraum dokumentierten, unmittelbar eine ärztliche Abklärung benötigenden Ereignisse (wie oben definiert) bei den eingeschlossenen Patienten ausgewertet.

### Dunkelziffer

Komplikationen erfordern sofortige und nachhaltige krankenhausmedizinische Interventionen, die Zeit binden. Dokumentation erfolgt nachrangig. Dies führt dazu, dass Komplikationen in Kliniken nur unvollständig erfasst werden. Um die daraus resultierende Dunkelziffer nichtdokumentierter Komplikationen zu schätzen, verglichen wir die Einträge in vier Erfassungsquellen (elektronisches klinisches Informationssystem, elektronische Medikamentendatenbank, papierbasierte Notfall- oder Sturzprotokolle und mündliche Qualitätschecks in der Frühbesprechung).

### Statistische Analyse

Die statistische Analyse erfolgte per JASP (Universität Amsterdam). Zunächst analysierten wir Anzahl und Art der krankenhausmedizinischen Ereignisse sowie deren Latenz zur Aufnahme in die Neurorehabilitationsklinik und zum zugrunde liegenden neurologischen Initialereignis. Für die Berechnungen der Latenzen wurden nur Komplikationen von Patienten berücksichtigt, deren gesamter Aufenthalt in der Neurorehabilitationsklinik in den Beobachtungszeitraum dieser Erhebung fiel, um Verzerrungen durch das Verpassen vor oder nach diesem Zeitraum auftretender Komplikationen zu vermeiden.

Eine logistische Regressionsanalyse prüfte dann, ob die Komplikationswahrscheinlichkeit eines Patienten systematisch von den folgenden Faktoren abhängt: Beobachtungsdauer (in Tagen, Kontrollvariable), Komorbidität (quantifiziert durch gewichteten Charlson-Index), Pflegeabhängigkeit (quantifiziert durch Barthel-Index bei Aufnahme in die Neurorehabilitationsklinik), Latenz zwischen Akutereignis und Aufnahme in die Neurorehabilitationsklinik und Alter. Diese Regressionsanalyse wurde separat für die Komplikation Stürze und alle anderen Komplikationsarten durchgeführt, da Stürze mit Mobilität und andere Komplikation mit Organfunktion assoziiert sind.

### Ethik

Diese prospektive Beobachtungsstudie wurde von der Ethikkommission der Medizinischen Fakultät der Heinrich-Heine-Universität positiv begutachtet (#6028R). Alle in die Analyse eingegangen Patienten haben ihr Einverständnis schriftlich bestätigt.

## Ergebnisse

### Charakteristika der untersuchten Patientenkohorte

**Figure 1:**
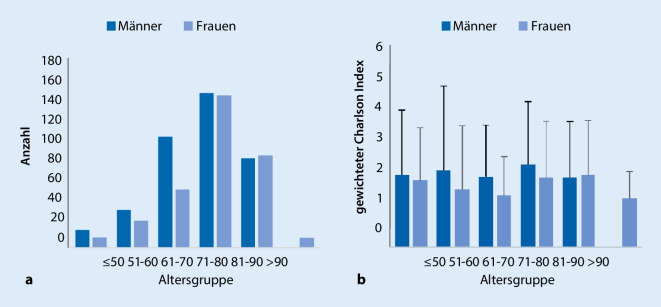

Die untersuchte Patientenkohorte umfasste 759 Patienten (410 Männer, 349 Frauen) mit einer Summe von 27.048 Behandlungstagen innerhalb des Beobachtungszeitraums. Die Mehrheit der Patienten (61 %) litt an einem ischämischen Schlaganfall und 16 % an einer Hirnblutung (siehe Tab. 1 für Häufigkeit anderer Hauptdiagnosen). Der Altersdurchschnitt lag bei 73 Jahren (±11 Jahre; siehe Abb. 1 für Verteilung), der gewichtete Charlson-Index lag im Schnitt bei 2,35 (±1,92) und das geschätzte Ein-Jahres-Mortalitätsrisiko bei 19 % (±11 %). Der Median der Latenz zwischen dem Akutereignis und Aufnahme in unsere Klinik betrug 24 Tagen (Mittelwert = 37 Tage).

**Table 1:** 

Diagnose	Prozent
Ischämischer Schlaganfall	61
Hirnblutung	16
PNP/CIP/GBS	6
Rückenmarkserkrankungen	4
Hirntumor	3
Globale Hypoxie	1
Morbus Parkinson	1
Multiple Sklerose	0.7
Schädel-Hirn-Trauma	0.5
Andere	8

*PNP* Polyneuropathie, *CIP* Critical-illness-Polyneuropathie, *GBS* Guillain-Barré-Syndrom

Zu Beginn der individuellen Beobachtungszeit befanden sich 27 % der Patienten in Phase B, 46 % in Phase C und 27 % in Phase D.

### Häufigkeit und Art der auftretenden Ereignisse

Im Beobachtungszeitraum traten 602 Ereignisse auf, die unmittelbarer ärztlicher Abklärung bedurften (Tab. 2). Diese verteilten sich auf 300 der beobachteten Patienten (40 %). Am häufigsten traten Stürze (*n* = 299), Harnwegsinfekte (*n* = 101), Fieber (*n* = 39), *Clostridium-difficile*-Enteritis (*n* = 33), Pneumonien (*n* = 26) und respiratorische Insuffizienz (*n* = 14) auf. Es wurden 6 Todesfälle (davon ein Suizid) verzeichnet. Bei 4 der Todesfälle war zuvor eine Therapiebegrenzung festgelegt worden. 47 % der Ereignisse traten bei sich zu diesem Zeitpunkt in Phase B befindlichen Patienten auf, 46 % der Ereignisse bei Patienten in Phase C und 7,5 % der Ereignisse trafen Patienten in Phase D.

**Table 2:** 

Ereignis	Anzahl	Prozent
Sturz	299	49
Harnwegsinfekt	101	17
Fieber	39	6,5
*Clostridium-difficile*-Enteritis	33	5,5
Pneumonie	26	4,3
Respiratorische Insuffizienz	14	2,3
Antibiose	7	1,2
Epileptischer Anfall	8	1,3
Herzrhythmusstörung	8	1,3
Gastrointestinale Blutung	7	1,1
Sepsis	6	1,0
Tod	6	1,0
Spondylodiszitis	5	0,8
Hämodynamische Instabilität	5	0,8
Lungenembolie	5	0,8
Wundinfektion	4	0,7
Gastroenteritis	3	0,5
Makrohämaturie	3	0,5
Akutes Abdomen	3	0,5
Reinfarkt	3	0,5
Durchfall	2	0,3
Erysipel	2	0,3
Akutes Nierenversagen	2	0,3
Psychomotorische Unruhe	2	0,3
Urosepsis	2	0,3
Abszess	1	0,2
Liquorzirkulationsstörung	1	0,2
Delir	1	0,2
Enzephalitis	1	0,2
Extrakranielle Blutung	1	0,2
Hirnabszess	1	0,2
Intrakranielle Blutung	1	0,2

**Figure 2:**
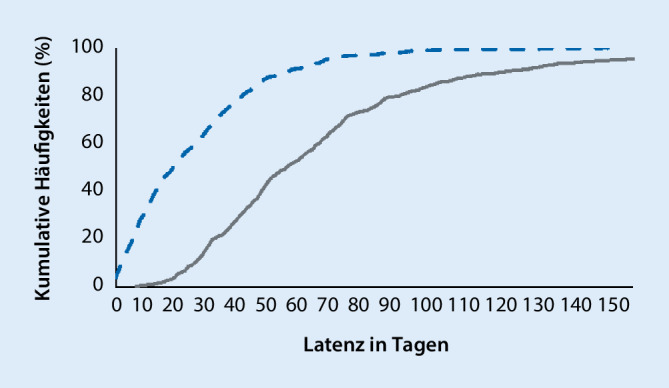

In der Berechnung der Latenzen der krankenhausmedizinischen Interventionsnotwendigkeit konnten 418 Ereignisse berücksichtigt werden. Die Latenz dieser Ereignisse zur Aufnahme in die Neurorehabilitationsklinik lag im Schnitt bei 22 Tagen (±21 Tage; Median = 16 Tage). Die mittlere Latenz zum zugrunde liegenden neurologischen Initialereignis lag bei 60 Tagen (±40 Tage; Median = 50 Tage). Abb. 2 zeigt die kumulative Häufigkeit der krankenhausmedizinischen Interventionsnotwendigkeiten im zeitlichen Verlauf.

**Figure 3:**
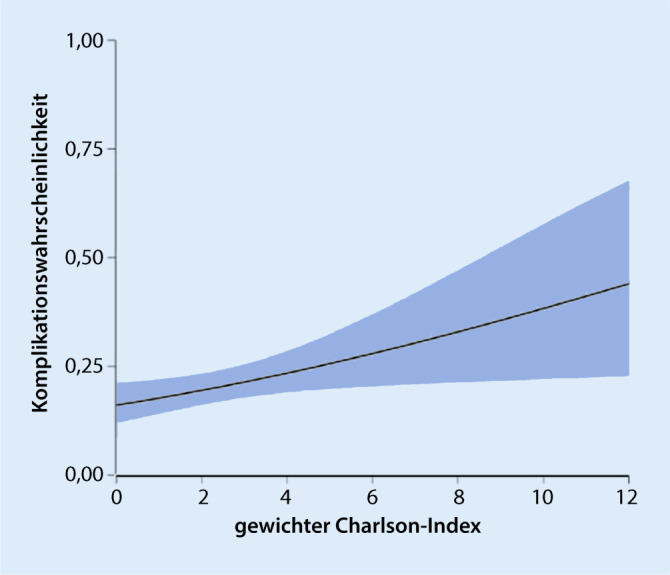

Die Wahrscheinlichkeit eines Patienten, eine (oder mehrere) krankhausmedizinische Komplikation(en) irgendeiner der o. g. Art außer Stürze zu erleiden, hing systematisch mit der Komorbidität und der Pflegebedürftigkeit bei der Aufnahme zusammen. Die Komplikationswahrscheinlichkeit stieg mit zunehmender Komorbidität (Β = 0,12, SE = 0,052, OR = 1,13, 95 %-CI = [1,02, 1,25], z = 2,28, *p* = 0,023) im Schnitt um 13 % pro zusätzlichem Punkt auf dem gewichteten Charlson-Index (Abb. 3) und sank mit der Selbsthilfefähigkeit (gemessen per Barthel-Wert; Β = −0,27, SE = 0,004, OR = 0,97, 95 %-CI = [0,97, 0,98], z = −6,47, *p* < 0,001) im Schnitt um 27 % pro 10 weniger Barthel-Index-Punkte. Wie erwartet, bestätigte sich zudem ein signifikanter Zusammenhang mit der Beobachtungsdauer eines Patienten in der Studie (Β = 0,012, SE = 0,004, OR = 1,01, 95 %-CI = [1,00, 1,02], z = 3,05, *p* = 0,002). Die Latenz zum zugrunde liegenden neurologischen Initialereignis (bei Aufnahme in die Neurorehabilitationsklinik) und Alter hatten hingegen keinen systematischen unabhängigen Einfluss auf die Komplikationswahrscheinlichkeit (*p* = 0,613 und *p* = 0,668). Die Wahrscheinlichkeit eines Patienten, einen Sturz (oder mehrere) zu erleiden, stieg mit zunehmender Pflegebedürftigkeit (Β = −0,02, SE = 0,004, OR = 0,98, 95 %-CI = [0,97, 0,99], z = −4,76, *p* < 0,001) im Schnitt um 18 % pro 10 weniger Barthel-Index-Punkte, zeigte jedoch keinen systematischen Zusammenhang mit Komorbidität (*p* = 0,71), Latenz (*p* = 0,474), oder Alter (*p* = 0,591; Beobachtungsdauer: Β = 0,007, SE = 0,004, OR = 1,01, 95 %-CI = [0,99, 1,01], z = 1,75, *p* = 0,08).

### Verlegungen

Bei 63 der o. g. Komplikationen (10,36 %) mussten Patienten in ein fachlich spezialisiertes Krankenhaus verlegt werden. In 8 Fällen war dies die Intensivstation der eigenen Krankenhausabteilung. In 32 Fällen waren dies Intensivstationen, internistische oder neurologische Abteilungen in anderen Krankenhäusern (Tab. 3) – in vielen Fällen, weil die eigene 30-Betten-Krankenhausabteilung vollständig belegt war.

**Table 3:** 

Fachabteilung	Anzahl
Innere	15
Intensiv	11
Neurologie	6
Chirurgie	5
Notfallambulanz	5
Neurochirurgie	3
Nicht dokumentiert	3
Urologie	3
Psychiatrie	2
Gefäßchirurgie	1
Orthopädie	1

### Dunkelziffer

Anhand der Komplikationsarten gehen wir dabei davon aus, dass 60 % der Interventionen in wenigsten zwei Quellen hätten dokumentiert werden müssen, fanden dies aber nur in 18 % der Fälle entsprechend einer Sensitivität von 30 %. Daraus folgt eine Verpassrate von wenigsten 45 % pro Quelle und über vier Quellen eine Dunkelziffer von wenigstens 4 % oder 33 Komplikationen.

## Diskussion

Hauptergebnis dieser Studie ist, dass bei knapp 40 % der Patienten in der neurologischen Anschlussrehabilitation eine oder mehr Komplikationen auftraten, die eine sofortige krankenhausmedizinische Intervention notwendig machten. Die Wahrscheinlichkeit eines Patienten, Komplikationen zu erleiden, stieg mit höherer Komorbidität und höherer Pflegebedürftigkeit.

Mit den hier angeführten Ereignissen sind nur die notfälligen krankenhausmedizinischen Interventionen beschrieben. Unberücksichtigt blieben weitere krankenhaustypische Maßnahmen wie die Anlage suprapubischer Blasenkatheter oder perkutaner endoskopischer Gastrostomiesonden, die bildgebungsbasierten Einstellungen von Ventilen für ventrikuloperitoneale Shunts oder intravenöse Behandlungen. Des Weiteren legen die Vergleiche der unterschiedlichen Dokumentationen nahe, dass wahrscheinlich mit zusätzlich wenigsten 4 % notfälliger krankenhausmedizinischer Interventionen gerechnet werden muss, die durch unser Vorgehen nicht erfasst wurden, weil die jeweiligen Ärzte sie möglicherweise als bereits normalen Arbeitsinhalt der neurologischen Anschlussrehabilitation ansahen und nicht dokumentiert haben. Angesichts des Alters und der Krankheitsschwere der Patienten in der neurologischen Anschlussrehabilitation erscheinen die Ergebnisse plausibel und passen zu Erhebungen von Komplikationsraten nach Schlaganfallakutbehandlung zwischen 44 und 84 % in anderen Sozialsystemen [3, 4, 7].

Eingewandt werden könnte, dass jede zweite Komplikation auf einen Sturz zurückging und sich diese auch in Pflegeheimen oder in der Häuslichkeit ereignen. Daraus zu folgern, dass Stürze in der Anschlussrehabilitation keine sofortige Interventionsbereitschaft verlangten, wäre allerdings ein Fehlrückschluss. Häufigkeit und Tragweite von Stürzen nach frischen Hirnverletzungen und unter oft antithrombotischer Therapie sind erheblich höher als im ambulanten Bereich. Das Gleiche gilt für die ca. 16 % Komplikationen infolge von Harnwegsinfekten. Denn bei entsprechender Indikation muss eine Antibiose unverzüglich gegeben werden, weil Verzögerungen die Mortalität signifikant erhöhen [8]. Dies gilt insbesondere bei Patienten, die infolge vorbestehender kognitiver Störungen nur eingeschränkt hinsichtlich Infektions- und Sepsissymptomen beurteilbar sind.

Insgesamt belegen die Ergebnisse dieser Erhebung daher die Vermutung, dass neurologische Anschlussrehabilitation der Sache nach Krankenhausbehandlung umfasst. Dies ist mittlerweile auch faktisch durch die obersten Planungsbehörden der Länder im Rahmen der COVID-19-Pandemie anerkannt. So wurden Neurorehabilitationszentren auf der Basis des § 22 Krankenhausgesetz als Einrichtungen bestimmt, in denen Patienten, die einer nicht aufschiebbaren akutstationären Krankenhausversorgung nach § 5 SGB V bedürfen, vollstationär behandelt werden können. Unsere Ergebnisse und das staatliche Handeln zeigen, dass sozialgesetzlicher Rahmen und Versorgungsrealität nicht (mehr) kongruent sind. Denn nach sozialgesetzlichen Vorgaben müssten nicht jederzeit ärztliches und anderes qualifiziertes Personal vor Ort sein. Dies wäre bei mehr als drei Komplikationen pro Tag in der untersuchten Einrichtung aber grob fahrlässig.

Zumindest in der Neurorehabilitation haben natürlich viele Kliniken mittlerweile durchgehende ärztliche Dienste und multidisziplinäre Fachkompetenz aufgebaut. Dies erhöht die Versorgungssicherheit für Patienten. Es geht aber zulasten von Ressourcen für genuine Rehabilitationsbehandlung. Die Kostenträger sparen derweil. Bei strenger Anwendung der Sektorengrenzen wäre in vielen der hier beschriebenen Fälle eine Verlegung in ein Krankenhaus indiziert gewesen. Geht man überschlagsweise von zwei Drittel der Fälle aus, berücksichtigt ferner zweimalige Transportkosten und eine externe Krankenhausbehandlung mit einem mittleren Relativgewicht von 0,75, so hätten sich pro Jahr Zusatzkosten für die Kostenträger von weit über 2 Mio. € alleine für die hier untersuchte Rehabilitationsklinik ergeben.

Wie kann der Inkongruenz zwischen der hier gefundenen krankenhausmedizinischen Versorgungsrealität der Neurorehabilitation und dem sozialgesetzlichen Rahmen begegnet werden? Neurologische Anschlussrehabilitation mit ihrem hohen Bedarf an krankenhausmedizinischen Interventionen könnte durch eine Zusammenlegung von Rehabilitationskliniken und Krankenhäusern geleistet werden. Dies würde dem Anspruch der Patienten auf medizinische Rehabilitation und ihren häufigen Bedarf an krankenhausmedizinischer Intervention gerecht werden. Diese Strukturen haben sich bereits an vielen Stellen entwickelt durch Umwandlung neurologischer Frührehabilitationseinheiten an Rehabilitationskliniken in Krankenhausabteilungen. Wir haben sie an anderer Stelle unter dem Begriff „integrierte Neurorehabilitation“ beschrieben [6]. Kern ist, dass in einem integrierten Zentrum eine Rehabilitationsabteilung jederzeit (24 h am Tag) auf Leistungen der Krankenhausabteilung zugreifen kann. Da das Krankenhausgesetz eine organisatorische und wirtschaftliche Trennung von Krankenhäusern und Rehabilitationskliniken verlangt, erfolgt diese Leistung als Konsil der Ärzte des Krankenhauses in der Rehabilitationsklinik. Während Zentren für integrierte Neurorehabilitation ursprünglich für die adäquate Versorgung neurologischer Frührehabilitationspatienten entstanden sind, könnten sie sich jetzt auch als probate, ja notwendige Struktur für die neurologische Anschlussrehabilitation erweisen.

Integrierte Neurorehabilitationszentren könnten die gefundenen Brüche zwischen Sozialgesetz und Versorgungsrealität aufheben und wieder Kongruenz herstellen. Allerdings bedürfte dies einer sektorenüberbrückenden staatlichen Versorgungsplanung, die gezielt den Aufbau von Krankenhausstrukturen in Neurorehabilitationskliniken unterstützt und so integrierte Neurorehabilitationszentren aufzubauen hilft. Leider wird aber auf Länderebene häufig noch Krankenhausbedarf isoliert von Rehabilitationsbedarf behandelt. Unsere Daten zeigen, dass dies im Falle der neurologischen Anschlussrehabilitation kritisch, wenn nicht gar fahrlässig ist. Daneben dürfte eine auf Krankenhausbetten verengte Versorgungsplanung auch teuer sein. Denn neurologische Anschlussrehabilitationskliniken ohne Möglichkeiten zur krankenhausmedizinischen Versorgung müssen Patienten niederschwellig in Krankenhäuser verlegen.

Im Gegensatz dazu haben mehrere Bundesländer, u. a. Bayern, bereits in den 1980er-Jahren eine spezifische neurorehabilitative Versorgungskonzeption entwickelt. Deren planungsrechtliches Kernelement ist: alle Phasen einschließlich der Krankenhausphase der neurologischen Rehabilitation unter einem Dach. Mittlerweile gibt es in Bayern flächendeckend 29 entsprechende Spezialeinrichtungen mit über 1200 Betten [2]. Dies garantiert Patienten Krankenhausbehandlungsmöglichkeit auch in der neurologischen Anschlussrehabilitation und sogar in den weiteren Phasen. Die Ergebnisse unserer Untersuchung zeigen, dass diese Versorgungskonzeption zukunftsfähig war und in Sache und Rechtsanspruch auch heutigen älteren, kränkeren und komplikationsgefährdeteren Patienten in der neurologischen Anschlussrehabilitation gerecht wird. Unsere Ergebnisse unterstreichen, wie wichtig es ist, diese oder vergleichbare Versorgungskonzeptionen in allen Bundesländern umzusetzen.

## Fazit für die Praxis

- Heutige Patienten in der neurologischen Anschlussrehabilitation sind kränker und komplikationsgefährdeter als zu Zeiten, in denen der rechtliche Rahmen für die Neurorehabilitation entwickelt wurde.
- Bei zwei von fünf heutigen Patienten muss während der Rehabilitation ein- oder mehrmals vor Ort und sofort krankenhausmedizinisch interveniert werden, sodass neurologische Anschlussrehabilitation der Sache nach Krankenhausbehandlung geworden ist.
- Sozialrechtliche Ansprüche und Versorgungsrealität können wahrscheinlich am besten durch integrierte Neurorehazentren, die Rehabilitations- und Krankenhausabteilungen umfassen, zur Deckung gebracht werden.
